# COVID19 detection in appendix of acute appendicitis in a child: a case report and review of literature

**DOI:** 10.1186/s40792-023-01618-7

**Published:** 2023-03-14

**Authors:** Jun Kono, Koichiro Yoshimaru, Toshiharu Matsuura, Akihiko Tamaki, Junkichi Takemoto, Shinya Matsumoto, Taeko Hotta, Kenichi Kohashi, Yoshinao Oda, Tatsuro Tajiri

**Affiliations:** 1 Department of Pediatric Surgery, Reproductive and Developmental Medicine, Faculty of Medical Sciences, Kyushu University, 3-1-1 Maidashi, Higashi-Ku, Fukuoka, 812-8582 Japan; 2 Department of Anatomic Pathology, Graduate School of Medical Sciences, Kyushu University, 3-1-1 Maidashi, Higashi-Ku, Fukuoka, 812-8582 Japan; 3 Division of Pediatric Surgical Oncology, National Cancer Center Hospital, 5-1-1 Tsukiji, Chuo-Ku, Tokyo, 104-0045 Japan; 4 Department of Clinical Chemistry and Laboratory Medicine, Kyushu University Hospital, 3-1-1 Maidashi, Higashi-Ku, Fukuoka, 812-8582 Japan

**Keywords:** Acute appendicitis, COVID-19, SARS-CoV-2, RT-qPCR, Pediatrics

## Abstract

**Background:**

Gastrointestinal symptoms are one of the most common presentations of Coronavirus disease-19 (COVID-19), even in children. Higher rates of complicated appendicitis have been demonstrated in the era of the COVID-19 outbreak, and it has been recently suggested that acute appendicitis may occur as a complication of COVID-19. However, the relationship between appendicitis and COVID-19 remains unclear.

**Case presentation:**

A 7-year-old male presented to the pediatric emergency department with 2 days’ history of lower abdominal discomfort and tenderness. On examination, his abdomen was distended with diffuse mild tenderness at the lower abdomen, which was aggravated by movement. He was also tested and was found to be positive for SARS-CoV-2. Computed tomography showed perforated appendicitis with a fecalith. The patient was admitted and laparoscopic appendectomy was successfully performed. Postoperatively, a minor intra-abdominal abscess was present, which successfully treated with antibiotics. Histopathology showed a markedly inflamed appendix with mucosal ulceration and transmural neutrophilic inflammation, which was consistent with phlegmonous appendicitis. Reverse transcription quantitative polymerase chain reaction using a surgically extracted appendix specimen revealed the presence of SARS-CoV-2 virus, which indicated a pathophysiological relationship between appendicitis and COVID-19.

**Conclusion:**

The present case will provide further understanding of pediatric patients with concomitant COVID-19 and acute appendicitis.

## Background

The WHO declared Coronavirus disease-19 (COVID-19), which is caused by severe acute respiratory syndrome Coronavirus 2 (SARS-CoV-2), a global pandemic; the COVID-19 outbreak soon affected the whole world from its first appearance of December 2019 [1, 2].

Gastrointestinal symptoms are one of the most common presentations of COVID-19 [3]. Acute appendicitis is known to be one of the most common pediatric emergencies requiring the abdominal surgery in childhood and is diagnosed in 1 to 8 percent of children who undergo urgent evaluation [4, 5]. It has been recently suggested that acute appendicitis may occur as a complication of COVID-19 [6–10]. However, information regarding the relationship between acute appendicitis and COVID-19 infection is currently limited [6–9]; thus, the relationship remains unclear. A deeper understanding about the clinical presentation of acute appendicitis in COVID-19-infected pediatric patients is needed.

We herein report the case of a 7-year-old pediatric patient with concomitant COVID-19 and acute appendicitis. To the best of our knowledge, the present report to provide secondary substantiation of the presence of SARS-CoV-2 virus from a surgically extracted appendix specimen by reverse transcription quantitative polymerase chain reaction (RT-qPCR) in a patient suffering from acute appendicitis [6]. The present case will provide further understanding of pediatric patients with concomitant COVID-19 and acute appendicitis.

## Case presentation

A 7-year-old Japanese boy with no significant past medical history was referred to the emergency department (ED) of our institution from a primary and emergency medical center due to suspected appendicitis. He had lower abdominal discomfort and tenderness, which had persisted for 2 days. Due to the atypical symptoms, he was treated with laxatives and observed at home. Regarding his family history, his father tested positive for SARS-CoV-2. At the ED, he was febrile (39.1 °C), slightly tachycardic (heart rate 101 beats per minute) and normotensive (102/61 mmHg), O_2_ saturation of 99% (room air), and his respiratory rate was 15 breaths per minute. His abdomen was distended with diffuse mild tenderness at the lower abdomen, which was aggravated by movement. According to his family history, he was also tested and was found to be positive for SARS-CoV-2 by RT-qPCR. His initial laboratory values were normal, except for a high white blood cell (WBC) count (9900/µL) with a left shift (83.4% neutrophil count), and an elevated C-reactive protein level (12.3 mg/dL).

**Fig. 1:**
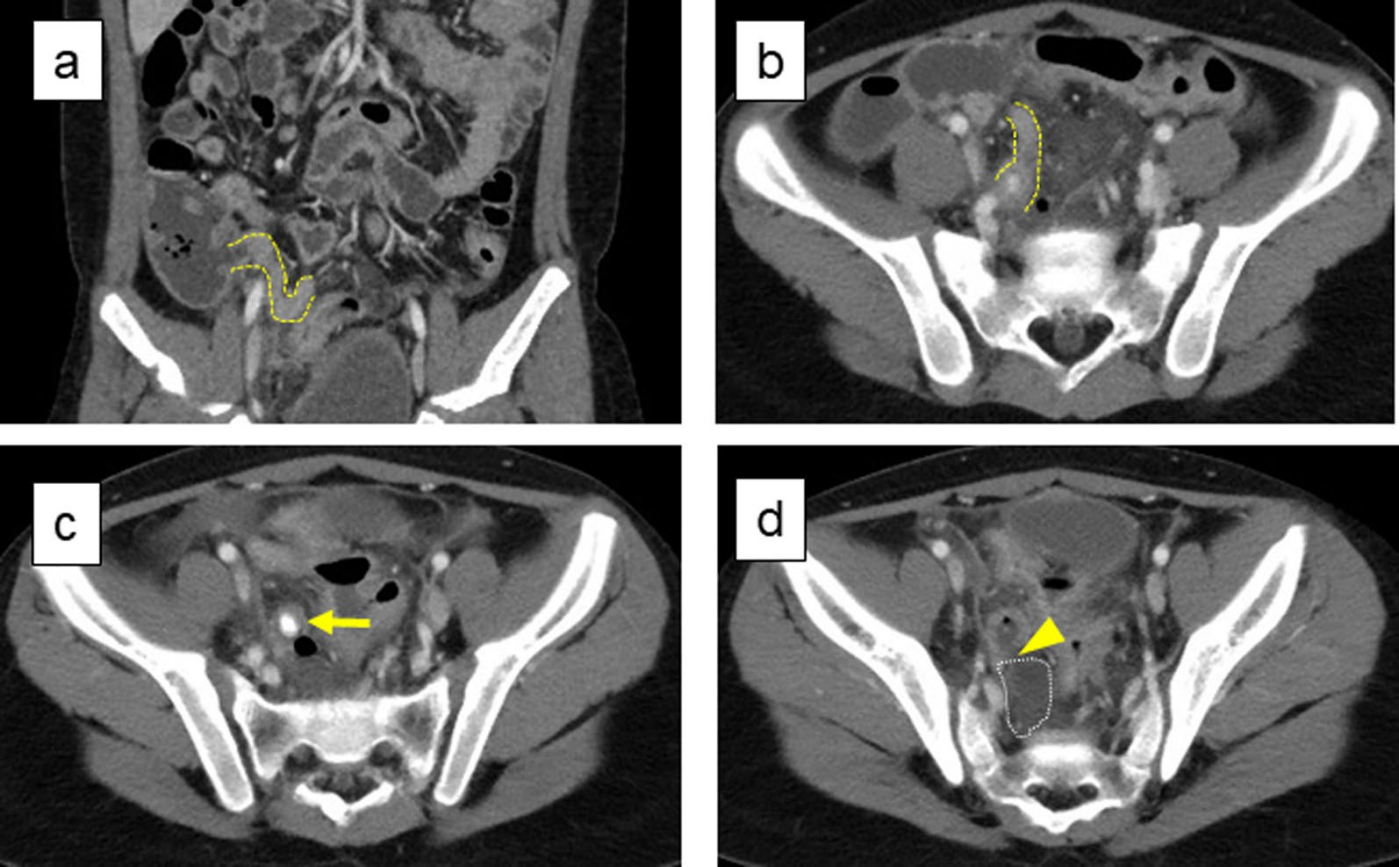
Enhanced computed tomography on admission. CT showed the swollen appendix with an intact appendicular base (**a**) and body (**b**), with a fecalith in the lumen (**c**), and perforation on the apex side (**d**), accompanied by fluid collection in the peri-appendiceal region (**d**). The yellow dotted line shows the appendicular body. The yellow arrow indicates a fecalith in the lumen. The yellow arrowhead shows the point of perforation. The white dotted line shows fluid collection in the peri-appendiceal region

Chest X-Ray showed no acute cardiopulmonary disease, and abdominal X-ray showed a slightly dilated bowel, indicating hypoperistalsis. On abdominal and pelvic ultrasonography, the appendix could not be visualized. Enhanced computed tomography (CT) of the abdomen/pelvis was performed to confirm the diagnosis and revealed a swollen appendix with an intact appendicular base (Fig. 1a) and the body (Fig. 1b), a fecalith in the lumen (Fig. 1c), and perforation on the apex side (Fig. 1d), accompanied by fluid collection in the peri-appendiceal region (Fig. 1d). These results were consistent with perforated appendicitis.

Laparoscopic appendectomy was favored at this point rather than non-operative management because of the patient’s stable physical condition, and perforated appendicitis with a fecalith. Additionally, the operating room was available because the day the patient visited our institution was a holiday and there were no scheduled surgeries.

All members of the surgical team, including the anesthesiologist and pediatric intensivists, wore enhanced personal protective equipment (PPE). The enhanced PPE included a surgical gown, head cover, face shield, two pairs of surgical gloves, shoe covers, and an N95 mask [11].

**Fig. 2:**
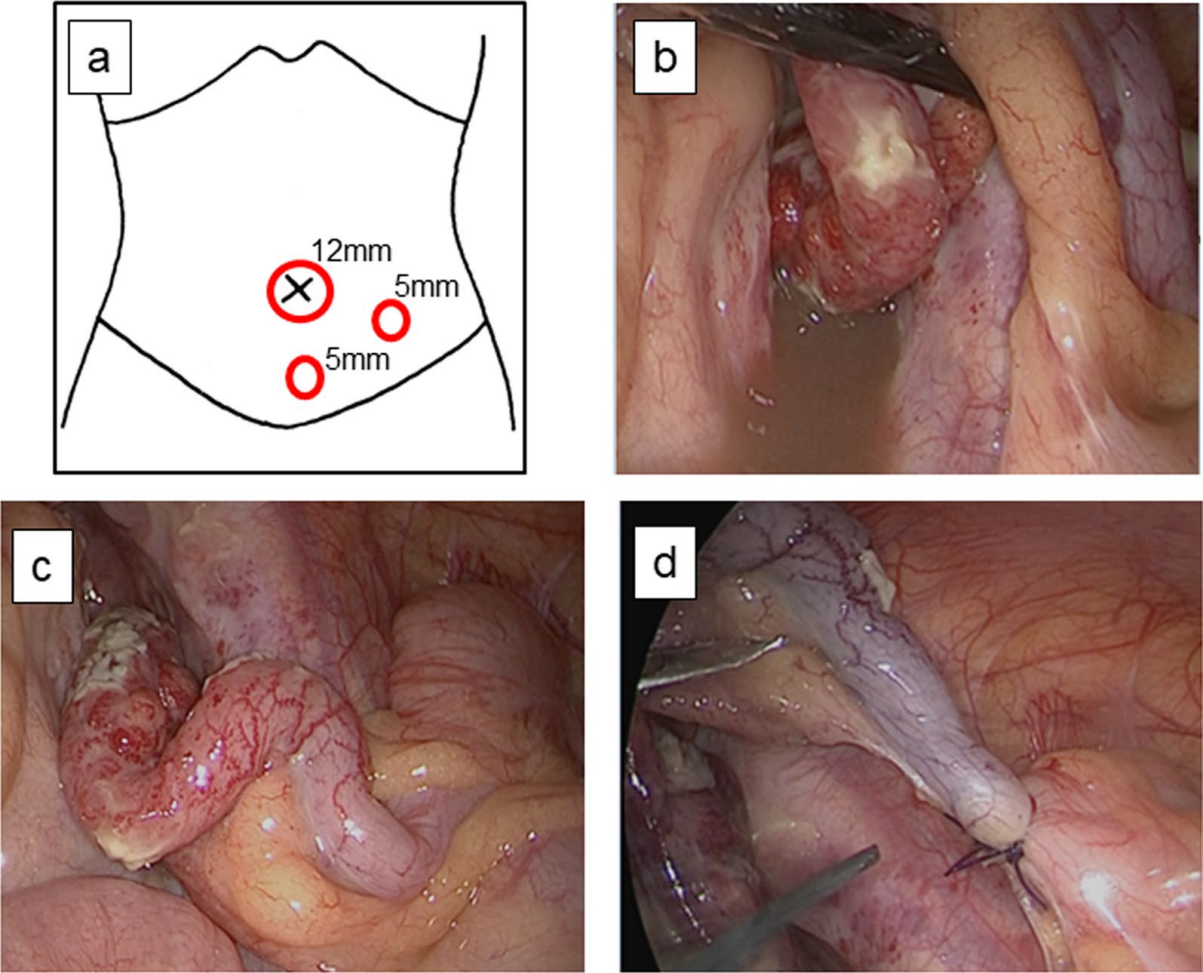
Intraoperative findings. **a** Schematic illustration of the port layout. **b** The swollen and perforated appendix with an intact base was adherent to the surrounding tissue, and purulent ascites was present in the rectovesical pouch. **c** The intact appendicular base was identified following careful adhesiolysis. **d** The mesentery of the appendix was divided, and the intact base of the appendix was ligated with double ENDOLOOPS®

The operation was performed as follows: a 12-mm port was used for the navel, and a 5-mm port was used for the upper pubic area and the left paramedian plane (Fig. 2a). Pneumoperitoneum was induced with an abdominal pressure of 8 mmHg at a CO_2_ flow rate of 4 L/min. The swollen and perforated appendix with the intact base was severely adherent to the surrounding tissue, and purulent ascites was present in the rectovesical pouch (Fig. 2b). After careful adhesiolysis (Fig. 2c), the mesentery of the appendix was divided, and the intact base of the appendix was ligated (Fig. 2d). The appendix was placed in a plastic bag and removed. The purulent ascites was taken for culturing and sensitivity testing. Abdominal lavage and drainage were carried out, and a closed drain was placed in the right iliac fossa via the rectovesical pouch. The operation lasted 93 min, anesthesia was applied for 165 min. Through the operation, we minimally used ultrasonic coagulation and incision device, and the adhesiolysis was mainly performed by a general peeling forceps to reduce the surgical mist because it is known to contain bio-aerosols with viable and non-viable cellular material that subsequently poses a risk of viral infection [12]. Awakening from anesthesia and oro-tracheal extubation were performed in a separated negative pressure intensive care unit to prevent the expansion of aerosol with PPE, and the patient was then moved to the infectious disease ward with a stable general condition.

Postoperatively, a minor intra-abdominal abscess that did not require surgical drainage was present. The culture from the appendiceal pus predominantly grew *Escherichia coli*, *Streptococcus constellatus*, and the post-operative blood culture grew *Bacteroides fragilis*. Then, we switched piperacillin-tazobactam to meropenem and metronidazole based on the results of sensitivity testing. The patient was discharged on postoperative day 17, after the disappearance of the intra-abdominal abscess. No respiratory symptoms related with COVID-19 infection (e.g., cough or sputum production) occurred postoperatively.

The patient received care from COVID-19-vaccinated staff members throughout the hospitalization period, and no staff members were infected.

**Fig. 3:**
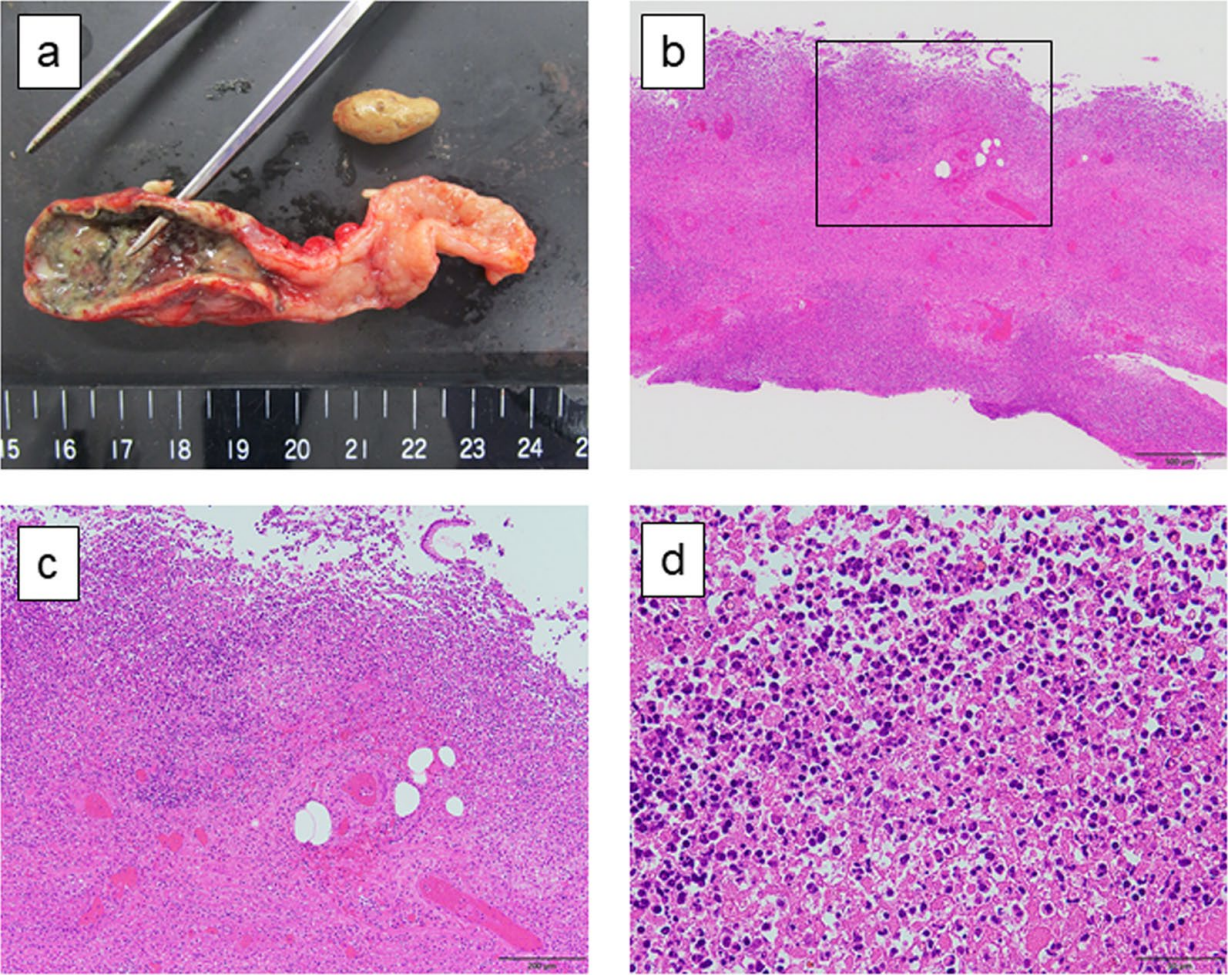
Histopathological findings of the extracted appendix. The pathological findings were consistent with phlegmonous appendicitis. The gross appearance revealed whole layer necrosis at the apical side of appendix, whereas the inflammation at the basal side of the appendix was limited. Fecaliths were found inside the appendix (**a**). **b**–**d** The markedly inflamed appendix with mucosal ulceration and transmural neutrophilic inflammation extending through the muscularis propria to the serosal surface. **c** Shows a high power field of the box in **b**. The scale bar indicates 500 µm in **b**; 200 µm in **c**; 50 µm in **d**. The magnification is ×40 in **b**; ×100 in **c**; ×400 in **d**

The pathological findings were consistent with phlegmonous appendicitis. The fecaliths were found inside the appendix (Fig. 3a). Histological sections with hematoxylin and eosin (H&E) staining showed a markedly inflamed appendix with mucosal ulceration and transmural neutrophilic inflammation extending through the muscularis propria to the serosal surface (Fig. 3b–d).

Next, to investigate the relationship between COVID-19 infection and appendicitis, we additionally performed RT-qPCR using an extracted cryopreserved appendix specimen. Briefly, the process was as follows: two tissue pieces from appendix (weight: approximately 15 mg) were homogenized, and total RNA was extracted. The detection of SARS-CoV-2 virus was performed using a SARS-CoV-2 Detection Kit-Multi (TOYOBO, Osaka, Japan). RT-qPCR was conducted, and was positive for SARS-CoV-2 (both the N1 and N2 regions).

## Discussion

In present study, the presence SARS-CoV-2 virus in a surgically extracted appendix specimen was demonstrated by RT-qPCR.

Several previous studies have reported a significantly increased prevalence of complicated acute appendicitis in the era of COVID-19 [13, 14]. The following reasons have been suggested: the time from the onset of symptoms is significantly longer during COVID-19 pandemic period; parental general condition and behavior changes during the COVID-19 outbreak; strained hospital resources; and that surgeons consider managing appendicitis with antibiotics alone whenever possible [14, 15].

We considered that pathophysiological mechanisms other than these clinical, environmental, and socioeconomic causes may underlie the increased frequency of complicated appendicitis. Acute appendicitis is known to be one of the most commonly reported causes of acute abdomen in children, the general pathophysiological cause of which appendicitis is obstruction of the lumen of the appendix by a fecalith or hyperplasic lymphoid follicle [14]. Also in present study, an appendiceal fecalith was present. Regarding the relationship between acute appendicitis and COVID-19, we could not determine how SARS-CoV-2 entered the appendix in the present study; however, we can suggest two possibilities: SARS-CoV-2 may have entered the gastrointestinal tract via oropharyngeal contamination or fecal–oral transmission may have occurred [16]. Ahmad et al. demonstrated positive RT-PCR results in rectal swabs of eight of ten pediatric patients, which remained detectable well after nasopharyngeal swabs turned negative, suggesting that the gastrointestinal tract may shed virus and that fecal–oral transmission may be possible [17]. SARS-CoV-2 is thought to use angiotensin converting enzyme 2 (ACE2) as a viral receptor, and ACE2 is highly expressed in the lung epithelium and enterocytes, especially in the small intestine [16, 18], which is also proven by immunohistochemistry [19, 20]. Once SARS-CoV-2 enters the gastrointestinal wall via ACE2 receptors, the inflammatory cells recruit and release pro-inflammatory cytokines, resulting in increased permeability, bacterial translocation, mucosal inflammation, and ACE2 dysfunction following post-obstructive appendicitis through the angioedema and stimulated hyperplasic lymphoid follicle [9, 21, 22]. Referring to these reports, we speculate that perforated acute appendicitis occurred in this case due to the following reasons: social predisposition in terms of family history and delayed medical examination, the original presence of an appendiceal fecalith, as well as the post-obstruction due to the angioedema and stimulated hyperplasic lymphoid follicle caused by penetration of SARS-CoV-2 into the appendix.

As the other pathophysiological interaction between appendicitis and COVID-19, the children with COVID-19 is reported to present with clinical features of appendicitis as part of the multisystem inflammatory syndrome in children (MIS-C) [7, 10, 23]. Although these authors described the causal relationship, Italian multicenter cohort study conversely showed no statistically significant association between MIS-C and appendicitis based on their multivariable analysis [24]; and Boybeyi‑Turer et al. [25] also concluded that careful evaluation and prompt diagnosis was important to avoid unnecessary surgical intervention since the involvement of terminal ileum in MIS-C may also be responsible for confusing the diagnosis with appendicitis. Thus, controversy still exists regarding the causal relationship between acute appendicitis, COVID-19 and MIS-C. Besides, because we couldn’t clinically figure MIS-C out in this case, the relationship between appendicitis and COVID-19 was unclear from this point of view.

Regarding the histopathological findings, pathological findings such as an extensive subserosal edema without mucosal ulceration, micro thrombi, fibrinoid necrosis of the blood vessels and perivascular lymphocytic inflammatory infiltration have been reported to occur in appendicitis associated with COVID-19 infection [6, 8], with more intense inflammation and vasculitis on the mesentery and the serosal side of the appendix in comparison to the appendiceal mucosa [7, 8]. However, in the present case, the entire appendiceal mucosa was markedly inflamed, making further histopathological examination of this inflammation difficult. The evaluation of the perivascular lesion, the mesoappendix as well as immunohistochemistry using anti-SARS-CoV-2 antibodies may lead to a more detailed histopathological evaluation [7].

In short, our findings consequently showed no definitive relationship between acute appendicitis and COVID-19 infection; however, the demonstration of the presence of SARS-CoV-2 by RT-qPCR may indicate a considerable relationship between acute appendicitis and COVID-19 infection. To further understand these correlations, careful sampling and assessment in a large series will be needed.

Finally, we summarized previously published case series in which SARS-CoV-2 was detected in the gastrointestinal tract (Table 1) [6, 19, 26, 27]. Gastrointestinal tissues have been tested in 4 studies thus far, and all studies tested for the presence of SARS-COV-2 using RT-PCR. Safari et al. reported a case series of 4 surgical patients, and SARS-COV-2 RNA was detected in the esophagus, duodenum, small intestine, and colon/rectum samples [26]. The rest of the studies were single-patient case reports: Xiao et al. [19] reported positive findings from multiple lesions using endoscopic biopsy. Ahmad et al. [6] and Goh et al. [27] demonstrated positive findings from resected appendix due to appendiceal lymphoid hyperplasia and acute appendicitis, respectively. Based on the previous publications and our study, we should pay attention to the presence of SARS-CoV-2 in biopsied or resected tissue when we process gastrointestinal specimens.

**Table 1:** Summary of previously published case series which detected SARS-CoV-2 in the gastrointestinal tract

Authors	Age	Sex	Method	Disease	Location						Clinical outcome
					Eso	Sto	Duo	Sma	Appe	Col/Rec	
Safari et al. [23]	75	F	Operation, LASC	GB empyema	Positive	N.S	N.S	N.S	N.S	Negative	Died
	39	M	Operation, resection	Intestinal perforation	Positive	N.S	N.S	Negative	N.S	Positive	Alive
	32	M	Operation, appendectomy	Acute appendicitis	Positive	N.S	N.S	N.S	Negative	Positive	Alive
	30	M	Operation, loop gastrojejunostomy	Perforated peptic ulcer	Positive	N.S	Positive	N.S	N.S	Positive	Died
Xiao et al. [19]	N.A	N.A	Endoscopy	N.A	Positive	Positive	Positive	N.S	N.S	Positive	N.A
Ahmad et al. [6]	28	M	Operation, appendectomy	Acute appendicitis	N.S	N.S	N.S	N.S	Positive	N.S	N.A
Goh et al. [10]	44	F	Operation, appendectomy	Appendiceal lymphoid hyperplasia	N.S	N.S	N.S	N.S	Positive	N.S	N.A
Present case	7	M	Operation, appendectomy	Acute appendicitis	N.S	N.S	N.S	N.S	Positive	N.S	Alive

*Eso* esophagus, *Sto* stomach, *Duo* duodenum, *Sma* small intestine, *Appe* appendix, *Col/Rec* colon and rectum, *LASC* laparoscopic cholecystectomy, *GB* gallbladder, *N.S* not sampled, *N.A* not available

## Conclusions

The relationship between appendicitis and COVID-19 isn’t fully understood, however our study might indicate that acute appendicitis can be considered as a complication of COVID-19. Children presenting gastrointestinal symptoms accompanied by COVID-19 should be closely followed to detect appendicitis.

## Data Availability

Not applicable.

## Abbreviations

RT-qPCR: Reverse transcription quantitative polymerase chain reaction
COVID-19: Coronavirus disease-19
ED: Emergency department
SARS-CoV-2: Severe acute respiratory syndrome Coronavirus 2
CT: Computed tomography
PPE: Personal protective equipment
MIS-C: Multisystem inflammatory syndrome in children
ACE2: Angiotensin converting enzyme 2
